# Modeling the Evolution of Rates of Continuous Trait Evolution

**DOI:** 10.1093/sysbio/syac068

**Published:** 2022-11-16

**Authors:** Bruce S Martin, Gideon S Bradburd, Luke J Harmon, Marjorie G Weber

**Affiliations:** Department of Plant Biology, Ecology, Evolution, and Behavior Program, Michigan State University, East Lansing, MI 48824, USA; Department of Ecology and Evolutionary Biology, University of Michigan, Ann Arbor, MI 48109, USA; Department of Biological Sciences, Institute for Bioinformatics and Evolutionary Studies (IBEST), University of Idaho, Moscow, ID 83843, USA; Department of Ecology and Evolutionary Biology, University of Michigan, Ann Arbor, MI 48109, USA

## Abstract

Rates of phenotypic evolution vary markedly across the tree of life, from the accelerated evolution apparent in adaptive radiations to the remarkable evolutionary stasis exhibited by so-called “living fossils.” Such rate variation has important consequences for large-scale evolutionary dynamics, generating vast disparities in phenotypic diversity across space, time, and taxa. Despite this, most methods for estimating trait evolution rates assume rates vary deterministically with respect to some variable of interest or change infrequently during a clade’s history. These assumptions may cause underfitting of trait evolution models and mislead hypothesis testing. Here, we develop a new trait evolution model that allows rates to vary gradually and stochastically across a clade. Further, we extend this model to accommodate generally decreasing or increasing rates over time, allowing for flexible modeling of “early/late bursts” of trait evolution. We implement a Bayesian method, termed “evolving rates” (*evorates* for short), to efficiently fit this model to comparative data. Through simulation, we demonstrate that *evorates* can reliably infer both how and in which lineages trait evolution rates varied during a clade’s history. We apply this method to body size evolution in cetaceans, recovering substantial support for an overall slowdown in body size evolution over time with recent bursts among some oceanic dolphins and relative stasis among beaked whales of the genus *Mesoplodon*. These results unify and expand on previous research, demonstrating the empirical utility of *evorates*. [cetacea; macroevolution; comparative methods; phenotypic diversity; disparity; early burst; late burst]

The rates at which traits evolve are markedly heterogeneous across the tree of life, as evidenced by the uneven distribution of phenotypic diversity across space, time, and taxa (e.g., Simpson, 1944; Brusatte et al., 2012; Reaney et al., 2020; Chartier et al., 2021). While understanding the drivers of such patterns can provide critical insights into macroevolutionary processes, a general consensus on what factors are most important in accelerating and decelerating trait evolution remain elusive (Chira et al., 2018). There is a vast, interconnected web of factors hypothesized to affect trait evolution rates, typically divided into extrinsic and intrinsic components. Extrinsic factors relate to the environment of an evolving lineage, commonly including aspects of biogeography like climate or habitat (e.g., Clavel and Morlon, 2017; Mihalitsis and Bellwood, 2019), as well as interactions with other species (e.g., Slater, 2015; Borstein et al., 2019; Drury et al., 2021). Intrinsic factors instead involve properties of the evolving lineage itself, including life-history attributes such as behavior or developmental traits (e.g., Muñoz and Bodensteiner, 2019; Fabre et al., 2020) and genetic features like trait heritability and effective population size (e.g., Arnold et al., 2008; Villar et al., 2014). The effects of all these variables are interrelated and depend on the particular traits being studied, further complicating matters (Cooper and Purvis, 2009; Muñoz et al., 2018; see also Donoghue and Sanderson, 2015).

Unfortunately, the evolutionary histories of many factors hypothesized to affect trait evolution rates are largely unobserved. Thus, methods testing for associations between rates and variables of interest must first estimate the history of the explanatory variables themselves (but see Hansen et al., 2022). This limits researchers to considering only a few, relatively simple hypotheses (Revell, 2013; Caetano and Harmon, 2019), causing trait evolution models to often underfit observed data (Pennell et al., 2015; Chira and Thomas, 2016; Chira et al., 2018). This underfitting generally oversimplifies inferred rate variation patterns and artificially increases statistical support for complex models which may imply spurious links between trait evolution rates and explanatory variables (May and Moore, 2020; see also Rabosky and Goldberg, 2015; Beaulieu and O’Meara, 2016). Thus, these “hypothesis-driven” approaches to modeling trait evolution should be integrated with “data-driven” approaches that agnostically model variation in trait evolution rates based on observed trait data alone. Such approaches can account for rate variation unrelated to some focal hypothesis, or even be used to generate novel hypotheses regarding what factors may have driven inferred rate variation patterns (Uyeda et al., 2018; May and Moore, 2020; see also Beaulieu and O’Meara, 2016).

Several data-driven methods for inferring trait evolution rates are already available and widely used (Eastman et al., 2011; Thomas and Freckleton, 2012; Rabosky et al., 2014; Pagel et al., 2022), but such methods generally work by splitting phylogenetic trees into subtrees and assigning a unique rate to each subtree (sometimes termed “macroevolutionary regimes”). These models implicitly assume trait evolution rates stay constant over long periods of time with sudden shifts in particular lineages. This mode of rate variation would be expected if rates are primarily influenced by only a few, discretely varying factors of large effect. However, this assumption could be problematic given the sheer number of factors hypothesized to affect trait evolution rates, as well as the fact that many of these factors vary continuously (Cooper and Purvis, 2009). If rates are instead affected by many factors, mostly with subtle effects, we would expect trait evolution rates to constantly shift in small increments over time within a given lineage, resulting in gradually changing rates over time and phylogenies. In other words, rates themselves would “evolve” and be similar, but not identical, among closely related lineages (i.e., phylogenetic autocorrelation; see Sakamoto and Venditti, 2018). By assuming that rates change infrequently, current data-driven methods likely oversimplify rate variation patterns, collapsing heterogeneous evolutionary processes into homogeneous regimes (but see May and Moore, 2020; Fisher et al., 2021). To this end, Revell (2021) recently developed a data-driven method that models trait evolution as gradually changing, but this method is limited in requiring *a priori* specification of how much trait evolution rates vary across the phylogeny. Further, the method offers no way to rigorously test whether lineages exhibit different rates (Revell, 2021).

Notably, some *hypothesis-driven* methods model trait evolution rates as gradually changing over time. However, such models most commonly assume that rates only follow a simple trend of exponential decrease or increase over time (Blomberg et al., 2003; but see Clavel and Morlon, 2017; Slater et al., 2017). In this context, declining trait evolution rates, or “early bursts” (EB), are often invoked as signatures of adaptive radiation (Harmon et al., 2010), while increasing trait evolution rates, or “late bursts” (LB), are sometimes linked to processes like character displacement (Weber et al., 2016; Skeels and Cardillo, 2019). Unfortunately, current methods lack statistical power to detect decreasing trends in rates when just a few lineages deviate from an overall EB pattern (Slater and Pennell, 2014). Essentially, by assuming a perfect correspondence between time and rates across all lineages, inference under these methods is misled by subclades exhibiting anomalously low- or high-trait evolution rates. New methods that explicitly model such “residual” rate variation may more accurately detect general trends in trait evolution rates by accounting for these anomalous lineages/subclades.

Here we develop a new, data-driven method that models trait evolution rates as gradually changing over time, ultimately resulting in stochastic, continuously distributed rates that are more similar among closely related lineages. We take advantage of recent developments in Bayesian inference and develop new strategies for efficiently estimating autocorrelated rates on phylogenetic trees while dealing with uncertain trait values, resulting in relatively fast, reliable inference. We call this method (and its corresponding software implementation) “evolving rates” or *evorates* for short. *Evorates* is both flexible and intuitive, allowing researchers to infer both how and where rates vary on a phylogeny. Through simulation, we demonstrate that *evorates* recovers accurate parameter estimates on ultrametric phylogenies spanning a range of sizes and that it is more sensitive and robust in detecting trends in trait evolution rates than conventional EB/LB models. We also use *evorates* to model body size evolution among extant whales and dolphins (order cetacea) and find evidence for declining rates of body size evolution and moderate rate heterogeneity in this clade, unifying and expanding on previous results (Slater et al., 2010; Slater and Pennell, 2014; Sander et al., 2021).

## Materials and Methods

*Evorates* uses comparative data on a univariate continuous trait to infer how trait evolution rates change over time as well as which lineages in a phylogeny exhibit anomalous rates. Here, comparative data refers to a fixed, rooted phylogeny with branch lengths proportional to time and trait values associated with its tips. We generally caution against using *evorates* with univariate ordinations of multivariate trait data such as principal component scores because ordination can bias rate inference from comparative data (Uyeda et al., 2015). *Evorates* is designed to work with raw trait measurements; both missing data and multiple trait values per tip are allowed (i.e., tips with 0 and >1 observations, respectively). In the case of averaged trait measurements, estimated mean trait values and standard errors can be used to specify normal priors on trait values at particular tips. The current implementation also allows for assigning raw trait measurements and priors to internal nodes as well, perhaps reflecting fossil data and/or strong prior beliefs, though we do not test this feature here. Conditional on these trait data, *evorates* uses Bayesian inference to estimate two key parameters governing the process of rate change: Rate variance, controlling how quickly rates diverge among independently evolving lineages, and a trend, determining whether rates tend to decrease or increase over time. When rate variance is 0, rates do not accumulate random variation over time and are constant across contemporaneous lineages. In this case, trait evolution follows the same exact process as expected under a conventional EB/LB model, with negative trends corresponding to EBs, no trend to Brownian Motion (BM), and positive trends to LBs. The method also infers branchwise rates, which are estimates of average trait evolution rates along each branch in the phylogeny, indicating which lineages exhibit unusually low or high rates.

### The Model

At its core, *evorates* works by extending a typical Brownian Motion (BM) model of univariate trait evolution to include stochastic, incremental changes in trait evolution rates, $\sigma^{2}$. Specifically, $\sigma^{2}$ follows a process approximating geometric BM (GBM) with a constant rate, meaning that $ln\left(\sigma^{2}\right)$ follows a homogeneous BM-like process. GBM is a natural process to describe “rate evolution” because it ensures rates stay positive and implies rates vary on a multiplicative, as opposed to additive, scale (Limpert et al., 2001; Gingerich, 2009). To render inference under this model tractable, we treat it as a hierarchical model with a trait evolution process dependent on the unknown—but estimable—branchwise rates, which are themselves dependent on a rate evolution process controlled by the estimated rate variance and trend parameters. The overall posterior probability (PP) of the model can be summarized as


$$\begin{array}{l} \begin{array}{lll} P(\bar{\sigma^{2}},\theta |x,\psi )\propto P(x|\psi ,\bar{\sigma^{2}})P(\bar{\sigma^{2}}|\psi ,\theta )P(\theta ) & \; & \end{array} \end{array}$$ (1)

where ψ is a phylogeny with e branches and n tips, $\bar{\sigma^{2}}$ is an e-length vector of branchwise rates, x is an n-length vector of trait values for each tip, and θ is a vector of parameters governing the rate evolution process. Cases with missing data and multiple trait values per tip are covered in a later section. In our notation, time is 0 at the root of the phylogeny and increases toward the tips. $P(x|\psi ,\bar{\sigma^{2}})$ is the likelihood of x given the trait evolution process, $P(\bar{\sigma^{2}}|\psi ,\theta )$ is the probability of branchwise rates given the rate evolution process, and P(θ) is the prior probability of the rate evolution process parameters. We explicitly estimate and condition likelihood calculations on branchwise rates (a type of “data augmentation”; see May and Moore, 2020) because the likelihood of the trait data while marginalizing over branchwise rates (i.e., P(x | ψ,θ)) does not follow a known probability distribution and would require complex, numerical approximations to compute. On the other hand, $P(x|\psi ,\bar{\sigma^{2}})$ follows a straightforward multivariate ­normal density:


$$\begin{array}{l} x∼MVN\left(\alpha ,C\right) \end{array}$$ (2)

where α is a vector of the trait value at the root of the phylogeny repeated n times and C is an n×n matrix. The entries of C are given by


$$\begin{array}{l} C_{i,j}=\sum_{k\in anc(i,j)}{\bar{\sigma^{2}}}_{k}t_{k} \end{array}$$ (3)

where t is an e-length vector of branch lengths, i and j are indices denoting specific tips, k is an index denoting a particular branch, and anc(i,j) is a function that returns all ancestral branches shared by i and j. Note that when branchwise rates are constant across the tree, $C_{i,j}$ is proportional to the elapsed time between the root of the phylogeny and the most recent common ancestor of i and j. Branchwise rates can be thought of as “squashing” and “stretching” the branch lengths of a phylogeny, such that certain lineages have evolved for effectively shorter or longer amounts of time, respectively.

Unfortunately, there is no general solution for calculating $P(\bar{\sigma^{2}}|\psi ,\theta )$ under a true GBM process (Lepage et al., 2007), so we instead use a multivariate log-normal approximation (e.g., Dufresne, 2004; Welch and Waxman, 2008) of the distribution of branchwise rates and calculate probabilities under this approximation. Briefly, this approximation decomposes branchwise rates into their expected values, β, determined solely by the trend parameter, and a “noise” component, γ, sampled from a multivariate normal distribution controlled by the rate variance parameter:


$$\begin{array}{l} \begin{array}{lll} ln(\bar{\sigma^{2}})\approx \beta +\gamma & \; & \end{array} \end{array}$$ (4)

Here, the noise component is approximate because it follows the distribution of geometric, rather than arithmetic, averages of trait evolution rates along each branch assuming there is no trend (i.e., $\bar{ln(\sigma^{2})}$ rather than $ln(\bar{\sigma^{2}})$; see Online Appendix for further details). The entries of β are given by


$$\begin{array}{l} \begin{array}{lll} \beta =ln(\sigma_{0}^{2})+\left\{ \begin{array}{ll} 0 & if\;\mu_{\sigma^{2}}=0\\ ln(|exp[\mu_{\sigma^{2}}\tau_{2}]-exp[\mu_{\sigma^{2}}\tau_{1}]|)-ln(|\mu_{\sigma^{2}}|)-ln(t) & if\;\mu_{\sigma^{2}}\neq 0 \end{array}\right. & \; & \end{array} \end{array}$$ (5)

where $ln\left(\sigma_{0}^{2}\right)$ is the estimated rate at the root of the phylogeny, $\mu_{\sigma^{2}}$ is the trend parameter, t is an e-length vector of branch lengths, and $\tau_{1}$ and $\tau_{2}$ are e-length vectors of the start and end times of each branch in the phylogeny (Blomberg et al., 2003). The entries of γ are given by


$$\begin{array}{l} \begin{array}{lll} \gamma ∼MVN\left(0,\sigma_{\sigma^{2}}^{2}D\right) & \; & \end{array} \end{array}$$ (6)

where 0 is a vector of 0s repeated e times, $\sigma_{\sigma^{2}}^{2}$ is the rate variance parameter, and D is an e×e matrix. The entries of D are given by


$$\begin{array}{l} \begin{array}{lll} D_{i,j}=\sum_{k\in anc(i,j)}t_{k}-\left\{ \begin{array}{ll} 2t_{i}/3 & if\;i=j\\ t_{i}/2 & if\;i\in anc(\;j,j)\\ t_{j}/2 & if\;j\in anc(i,i)\\ 0 & if\;i\neq j,i\text{⧸}\in anc(\;j,j),j\text{⧸}\in anc(i,i) \end{array}\right. & \; & \end{array} \end{array}$$ (7)

where i, j, and k are all indices denoting branches and anc(i,j) is a function that returns all ancestral branches shared by i and j (Devreese et al., 2010, see Online Appendix for further details). Overall, this approximation closely matches the distribution of branchwise rates obtained via fine-grained simulations of GBM on phylogenies under plausible parameter values and is negligibly different from other computationally efficient approximations (e.g., Thorne et al., 1998; Lartillot and Poujol, 2011; Revell, 2021; Figs. S3–S16; Tables S2–S4). We prefer this approximation because it is convenient to work with and directly focuses on estimating branchwise rates rather than rates at the nodes of the phylogeny, which is what other strategies focus on.

Under this approximation, the final expression for the PP is


$$\begin{array}{l} \begin{array}{ll} P(ln(\bar{\sigma^{2}}),\alpha ,ln(\sigma_{0}^{2}),\sigma_{\sigma^{2}}^{2},\mu_{\sigma^{2}}|x,\psi )\propto & \frac{exp[-\frac{1}{2}(x-\alpha {)}^{\prime }C^{-1}(x-\alpha )]}{\sqrt{{\left(2\pi \right)}^{n}|C|}}\\ & \frac{exp[-\frac{1}{2}(ln(\bar{\sigma^{2}})-\beta {)}^{\prime }{\left(\sigma_{\sigma^{2}}^{2}D\right)}^{-1}(ln(\bar{\sigma^{2}})-\beta )]}{\sigma_{\sigma^{2}}\sqrt{{\left(2\pi \right)}^{e}|D|}}\\ & P(\alpha ,ln(\sigma_{0}^{2}),\sigma_{\sigma^{2}}^{2},\mu_{\sigma^{2}}) \end{array} \end{array}$$ (8)

### Model Implementation

*Evorates* estimates the posterior distribution of parameters given a phylogeny and associated trait data via Hamiltonian Monte Carlo (HMC) using the probabilistic programming language Stan, interfaced through R (Carpenter et al., 2017; Stan Development Team, 2020, 2021). Unlike conventional Markov Chain Monte Carlo algorithms like Metropolis-Hastings samplers, HMC uses derivatives and physics simulations to efficiently explore posterior distributions, which is particularly helpful for complex, high-dimensional posteriors (see Neal, 2011; Hoffman and Gelman, 2014, for further information). To optimize sampling efficiency and avoid numerical issues, *evorates* estimates branchwise rates with an uncentered parameterization (Betancourt and Girolami, 2013) and marginalizes over unobserved trait values at the root and tips of the tree (Freckleton, 2012; Hassler et al., 2020). Under an uncentered parameterization, the HMC algorithm does not directly estimate branchwise rates, but instead estimates the distribution of e-independent standard normal random variables, z, which are transformed to follow the distribution of branchwise rates:


$$\begin{array}{l} ln(\bar{\sigma^{2}})=\sigma_{\sigma^{2}}Lz+\beta \end{array}$$ (9)

where L is lower triangular Cholesky factorization of D (i.e., $D=LL^{\prime }$; see Equation (7)). This parameterization is particularly efficient because it avoids having to repeatedly manipulate D to calculate $P(ln(\bar{\sigma^{2}})|\psi ,ln(\sigma_{0}^{2}),\sigma_{\sigma^{2}}^{2},\mu_{\sigma^{2}})$.

*Evorates* also uses Felsenstein’s pruning algorithm for quantitative traits to marginalize over the trait value at the root of the phylogeny and avoid repeatedly inverting C when calculating $P(x|ln(\bar{\sigma^{2}})$ (Felsenstein, 1973; Freckleton, 2012; Caetano and Harmon, 2019). To simplify the pruning algorithm implementation, any multifurcations in the phylogeny are converted to a series of bifurcations by adding additional “pseudo-branches” of length 0. This procedure does not alter the resulting likelihood calculations (Felsenstein, 2008), and our implementation does not estimate branchwise rates along pseudo-branches because these rates do not affect the likelihood of the observed trait data.

### Accommodating Missing Data and Multiple Observations

Incorporating uncertainty in observed trait values in comparative studies is especially important for methods that model trait evolution rate variation because measurement error can inflate estimates of evolutionary rates, particularly in young clades (Felsenstein, 2008). To prevent such biases, *evorates* generally treats the mean trait values at the tips, x, as unknown parameters. We marginalize over x given raw trait measurements, y (potentially including 0 or >1 observations for some tips), and “tip error” variances for each tip, $\sigma_{y}^{2}$. While we use the term “raw” trait measurement for clarity, the data provided for certain tips could instead be the mean of a normal prior on the trait value. Entries of $\sigma_{y}^{2}$ for such tips may be fixed to an associated variance for the prior. All other entries of $\sigma_{y}^{2}$ are treated as unfixed, free parameters. To render the model more tractable, we assume tip error variance is constant across all tips with unfixed variance.

To marginalize over the mean trait values at the tips, we modify the initialization of Felsenstein’s pruning algorithm (Felsenstein, 1973). Prior to pruning, we assign each tip the expectation and variance of its mean trait value given its raw trait measurements. We then calculate each tip’s partial likelihood from contrasts between its associated raw trait measurements given its error variance, $\sigma_{y,i}^{2}$. Assuming the raw trait measurements are independently sampled from a normal distribution with variance $\sigma_{y,i}^{2}$, the mean trait value’s expectation is simply the mean of the raw trait measurements, ${\bar{y}}_{i}$, and its variance is given by $\sigma_{y,i}^{2}/m_{i}$, where $m_{i}$ is the number of raw trait measurements (Felsenstein, 2008). Note that if there are no trait measurements for a particular tip (i.e., $m_{i}=0$), the expectation of that tip’s true trait value is undefined with infinite variance (Hassler et al., 2020).

Because there are no contrasts for tips with one or fewer raw trait measurements, the partial likelihood associated with these tips is 1. Otherwise, we can derive a general formula for the partial likelihood by considering each tip as a small subtree and applying Felsenstein’s pruning algorithm. Specifically, each tip is treated as a star phylogeny consisting of $m_{i}$ “sub-tips” of length $\sigma_{y,i}^{2}$, with trait values $y_{i}$ (Felsenstein, 1973, 2008):


$$\begin{array}{l} P(y_{i}|\sigma_{y,i}^{2})=\prod_{k=1}^{m_{i}-1}\frac{\sqrt{k}}{\sigma_{y,i}\sqrt{2\pi (k+1)}}exp\left[-\frac{k}{2(k+1)}{\left(\frac{y_{i,k+1}-\bar{y_{i,1:k}}}{\sigma_{y,i}}\right)}^{2}\right] \end{array}$$ (10)

where i denotes a particular tip, $y_{i}$ is a vector of $m_{i}$ raw trait measurements for tip i, $\sigma_{y,i}^{2}$ is the tip error variance for tip i, and $\bar{y_{i,1:k}}$ is the mean of measurements 1 through k in the vector $y_{i}$.

After initializing all tips in the phylogeny, Felsenstein’s pruning algorithm can be applied normally, iterating over the internal nodes from the tips toward the root (e.g., Felsenstein, 1973; Freckleton, 2012; Caetano and Harmon, 2019). The presence of missing data, however, will cause some calculations to involve nodes with undefined expected trait values and infinite variance. Note that these “data-deficient” nodes do not contribute information to the expectation and variance of the trait value at their ancestral nodes. Thus, if both nodes descending from some focal node are data deficient, the focal node will also be data deficient, with undefined expectation and infinite variance. Otherwise, if only one descendant node is data deficient, the expectation and variance of the trait value at the focal node is solely determined by the descendant node that is *not* data deficient. Let the descendant, non-data deficient node have expected trait value and variance $\hat{x_{i}}$ and $\sigma_{\hat{x_{i}}}^{2}$, respectively, and be connected to the focal node by a branch of length $t_{i}$ with branchwise rate $\bar{\sigma_{i}^{2}}$. The focal node’s expected trait value and variance will be $\hat{x_{i}}$ and $\sigma_{\hat{x_{i}}}^{2}+\bar{\sigma_{i}^{2}}t_{i}$, respectively. Whether one or both descendant nodes are data deficient, there is no contrast associated with the focal node and the corresponding partial likelihood is 1.

In the case of univariate traits, tips with missing data have no effect on the likelihood of trait data or parameter inference. However, by including missing data, one can estimate posterior distributions of the unobserved trait values at these tips (Goolsby, 2017; Hassler et al., 2020). *Evorates* already includes functionality for sampling from the posterior distribution of trait values at all nodes and tips in a phylogeny given a fitted model. The inclusion of additional branches could theoretically affect the inferred rate evolution process because our GBM approximation improves along shorter branches. However, inference using *evorates* is robust to whether rate evolution is simulated under our GBM approximation or a true GBM process (Figs. S12 and S16; Tables S2–S4), suggesting such effects are too minor to have practical consequences.

### Priors

Despite their popularity, flat and uninformative priors tend to result in fat-tailed posteriors that explore unrealistic regions of parameter space, and Bayesian statisticians have increasingly advocated for the use of at least weakly informative priors in recent years (Lemoine, 2019). We follow this advice, choosing default priors for *evorates* that modestly regularize parameter estimates, promoting conservative inferences (i.e., little rate heterogeneity) while still allowing for a wide range of evolutionary dynamics. We also conducted a prior sensitivity study to document the impact of priors on inference using *evorates* (Figs. S22–S28; Tables S8–S19). Overall, *evorates* is fairly robust to alternate prior specifications, provided that priors are not overly informative, and the default priors appear adequate under a variety of conditions.

By default, a normal prior with mean 0 and standard deviation 10/T is placed on the trend parameter ($\mu_{\sigma^{2}}$), while a Half-Cauchy prior with scale 5/T is placed on rate variance ($\sigma_{\sigma^{2}}^{2}$), where T is the height of the phylogeny. These priors are quite liberal: a trend of 10/T corresponds to a $e^{10}∼$ 20,000-fold change in trait evolution rates over the timespan of a phylogeny, and data simulated with a rate variance of 5/T on random trees with 50 tips or more (generated using the R package *ape* version 5.6-2; Paradis and Schliep, 2019) typically yield branchwise rates spanning 2–4 orders of magnitude. Of course, researchers may increase or decrease the standard deviation/scale of these priors if a phylogeny spans an especially long or short timescale, respectively. To penalize tip error variance ($\sigma_{y}^{2}$) estimates that are large relative to the scale of the observed trait data, a half-Cauchy prior with scale $\sigma_{raw}^{2}/2$ is placed on tip error variance, where $\sigma_{raw}^{2}$ is the variance of the trait data.

It is somewhat more challenging to pick a default prior for the rate at the root ($\sigma_{0}^{2}$) because this parameter depends on both the timescale of the phylogeny and scale of the observed trait data. By default, a log-normal prior with location $ln\left(\sigma_{raw}^{2}/T\right)$ and scale 10 is placed on the root rate. This prior is designed to regularize root rate estimation by roughly centering on trait evolution rates that could give rise to the observed trait data with little rate heterogeneity. Notably, decreasing and increasing trends will generally shift the location of this default prior downward and upwards, respectively, relative to the true root rate. While more complex schemes for choosing a root rate prior (perhaps based on phylogenetic independent contrasts) could help mitigate this issue, we wanted to keep default prior settings as simple and transparent as possible. As a rule of thumb, the scale of the root rate prior should be roughly equal to the maximum plausible change in trait evolution rates over the timespan of a phylogeny. The default scale of 10, corresponding to a $e^{10}∼$ 20,000-fold change in rates, is quite liberal and should suffice for most purposes. In any case, we encourage researchers to alter the root rate prior to reflect biologically plausible trait evolution rates when such information is available.

### Hypothesis Testing

We agree with other macroevolutionary biologists advocating for greater focus on interpreting parameter estimates and effect sizes inferred by comparative models (e.g., Beaulieu and O’Meara, 2016). Nonetheless, assessing statistical support for particular hypotheses remains important for biologically interpreting fitted models—particularly complex models with many parameters. In the context of *evorates*, we focus on two main hypotheses: 1) that significant rate heterogeneity, independent of any trend, occurred over the history of a clade ($\sigma_{\sigma^{2}}^{2}>0$), and 2) rates generally declined or increased over time (i.e., $\mu_{\sigma^{2}}\neq 0$). Both hypotheses could be tested by fitting additional models with constrained rate variance and/or trend parameters and comparing among unconstrained and constrained models using Bayes factors. However, Bayes factor estimation requires additional, time-consuming computation. Thus, we developed alternative approaches that only require the posterior samples of a fitted, unconstrained model.

We use the PP that $\mu_{\sigma^{2}}>0$ to test for overall trends in rates. If the PP is 0.025 or less, we can conclude that there is substantial evidence that rates declined over time, and vice versa if the PP is 0.975 or above. This corresponds to a two-tailed test with a critical value of 0.05. For rate variance, we instead use Savage–Dickey (SD) ratios because rate variance is bounded at 0 and the PP that $\sigma_{\sigma^{2}}^{2}>0$ will always be 1. SD ratios are ratios of the posterior to prior probability density at a particular parameter value corresponding to a null hypothesis. If this ratio is sufficiently less than 1, the data have “pulled” prior probability mass away from the null hypothesis, suggesting that the null hypothesis is likely incorrect. In general, a ratio of 1/3 or less is considered substantial evidence against the null hypothesis (Kass and Raftery, 1995). We use log spline density estimation implemented in the R package *logspline* (version 2.1.16) to estimate the PP density at $\sigma_{\sigma^{2}}^{2}=0$ (Stone et al., 1997; Wagenmakers et al., 2010).

Researchers may also wish to identify lineages evolving at anomalous rates. The most straightforward method to do so is to calculate the PP that branchwise rates are greater than some “background rate,” analogous to the approach for trends. In this paper, we define the background trait evolution rate as the geometric mean of branchwise rates, weighted by their relative branch lengths. Rates are generally distributed with long right tails (Gingerich, 2009), particularly under our model whereby rate evolution follows a GBM-like process. Geometric means are less sensitive than arithmetic means to extremely high, outlier rates associated with these long tails, and are thus better suited for rate comparisons. In the presence of a strong trend, only the oldest and youngest lineages will generally exhibit anomalous rates, rendering anomalous rate detection redundant with trend estimation. Thus, we define a helpful branchwise rate transformation, called “detrending,” which further facilitates the interpretation of *evorates* results. Specifically, branchwise rates are detrended prior to calculating background rates and posterior probabilities by subtracting β from branchwise rates on the natural log scale (see Equation (5)). These detrended rates yield a new set of transformed parameters, branchwise rate deviations, $ln(\bar{\sigma_{dev}^{2}})$, defined as the difference between detrended branchwise rates and the background detrended rate on the natural log scale. When the PP $ln(\bar{\sigma_{dev}^{2}})>0$ for a given branch is less than 0.025 or greater than 0.975, we can conclude that trait evolution is anomalously slow or fast along that branch, respectively, given the overall trend in rates through time. While we focus on comparing detrended branchwise and background rates based on geometric means in the current paper, we note that *evorates* can also compare untransformed branchwise and background rates based on either geometric or arithmetic means per user specifications.

Additionally, users may also calculate background trait evolution rates for subsets of branches in a phylogeny, such that rates for specific lineages and/or subclades can be estimated and compared. Some caution, however, is warranted in first identifying lineages exhibiting anomalous rates and then testing for significant differences among them, as this could increase the risk of spuriously detecting rate differences. This potential issue is not unique to *evorates* and applies to any data-driven phylogenetic comparative method designed to identify shifts in evolutionary processes. In practice, we recommend users mainly focus on interpreting comparisons between branchwise rates and the overall background rate, calculating background rates for branch subsets only to effectively summarize and communicate model results. Of course, it is also perfectly reasonable to compare rates among specific lineages and/or subclades when these comparisons are planned prior to model fitting and/or have biological justification (e.g., comparing background rates among lineages that vary in some factor hypothesized to affect trait evolution rates).

Notably, relationships among Bayes factors, posterior probabilities, and frequentist *p*-values are not necessarily straightforward and depend on sample size, priors, and posterior distribution shape, among other factors (Held and Ott, 2018; Wagenmakers et al., 2022). The hypothesis testing procedures we propose and test here are essentially useful heuristics developed to guide researchers in interpreting models fit through *evorates*, and these heuristics are not formally equivalent to conventional significance testing under a frequentist framework. Nonetheless, we use terms like “hypothesis testing,” “null hypothesis,” and “significance” in describing and analyzing the performance of these heuristics for ease of communication.

### Simulation Study

To test the performance and accuracy of *evorates*, we applied it to continuous trait data simulated under the model of inference. We simulated data under all combinations of no, low, and high rate variance ($\sigma_{\sigma^{2}}^{2}=0,3,6$) and decreasing, constant, and increasing trends ($\mu_{\sigma^{2}}=-4,0,4$), for a total of nine trait evolution scenarios. We picked these values to simulate data that appeared empirically plausible and represented a range of different trait evolution dynamics. Note that when the rate variance is 0, the resulting simulations evolve under EB, BM, or LB models of trait evolution depending on the trend parameter. We simulated traits evolving along ultrametric, pure-birth phylogenies with 50, 100, and 200 tips generated using the R package *phytools* (version 1.0-1; Revell, 2012) to assess the effect of increasing sample size on model performance. While *evorates* can be applied to non-ultrametric trees, we focus on ultrametric trees here to render the simulation study more manageable. We simulated 10 phylogenies and associated trait data for each trait evolution scenario and phylogeny size for a total of 270 simulations. In all cases, phylogenies were rescaled to a total height of 1, ensuring the effect of parameters remained consistent across replicates. All simulations were simulated with a trait and log rate value of 0 at the root. Because we focused on the estimation of branchwise rate, rate variance, and trend parameters, we simulated trait data with only one observation per tip and no tip error.

To quantitatively assess the simulation study results, we calculated the median absolute error (MAE), breadth, and coverage of marginal posterior distributions for rate variance and trend parameters. Here, MAE is the median absolute difference between posterior samples and their corresponding true, simulated value, such that larger MAEs are associated with less accurate posteriors. We prefer *median* to *mean* absolute error because the former metric is less influenced by posterior precision and more directly reflects variation in posterior accuracy. Breadth refers to the width of the 95% equal-tailed interval (i.e., a type of credible interval [CI] that spans from the 2.5% to 97.5% posterior quantiles, hereafter simply termed CIs) and measures posterior precision, with smaller breadths corresponding to more precise (though not necessarily accurate) posteriors. Lastly, coverage is a binary metric equal to one when the true value falls within the 95% CI and zero otherwise. For branchwise rate parameters, we averaged the MAEs, breadths, and coverage of all branchwise rate marginal posterior distributions (on the natural log scale) for each model fit. Additionally, we calculated the statistical power and false positive error rate (i.e., type I error rate, hereafter error rate) of *evorates* for detecting significant rate variance and decreasing/increasing trends. Due to the continuous nature of branchwise rates, we assessed power and error rates for detecting anomalous branchwise rates by calculating the proportion of times a branch is detected as exhibiting anomalously slow or rapid trait evolution rates across different values of true branchwise rate deviations.

### Empirical Example

We applied *evorates* to model body size evolution in extant cetaceans using a recently estimated timetree of both fossil and extant cetaceans (Lloyd and Slater, 2021), pruned to consist of 88 extant species (we excluded 1 extant species, *Balaenoptera brydei*, due to its uncertain taxonomic status; see Constantine et al., 2018), and associated trait data on log-transformed maximum female body lengths for each species. Most body length data were compiled in a previous comparative study, but we supplemented these data with published measurements for an additional 15 species (Table S1). We chose this example because previous research detected notable signatures of declining body size evolution rates over time in this clade, despite conventional model selection failing to yield support for an EB model of trait evolution. This puzzling result seems primarily due to a few recently evolved lineages exhibiting unusually rapid shifts in body size (Slater et al., 2010; Slater and Pennell, 2014; see also Sander et al., 2021). While previous work used a mix of simulation and outlier detection techniques to arrive at this conclusion, we predicted that our method would identify these patterns in a more cohesive modeling framework.

### HMC Configuration and Diagnostics

When fitting models to simulated and empirical data, we ran four HMC chains consisting of 3,000 iterations. After discarding the first 1,500 iterations as warmup and checking for convergence, chains were combined for a total of 6,000 HMC samples for each simulation. We repeated this procedure while constraining the rate variance parameter to 0 to see if our method could detect trends in trait evolution rates with more power than conventional EB/LB models. We set tip error for the simulation study to 0 *a priori* because we do not focus on the inference of this parameter here, though we did allow the method to estimate tip error in the empirical example. For each model fit, chains mixed well (greatest $\hat{R}\approx 1.013$) and achieved effective sample sizes of at least 3,000 for every parameter. Divergent transitions, a feature of HMC which can be indicative of sampling problems, were relatively rare, with only six simulation model fits exhibiting 1–3 divergent transitions. Overall, diagnostic tests suggested all HMC chains converged and sampled posterior distributions thoroughly.

## Results

### Performance of Method

**Figure 1.**
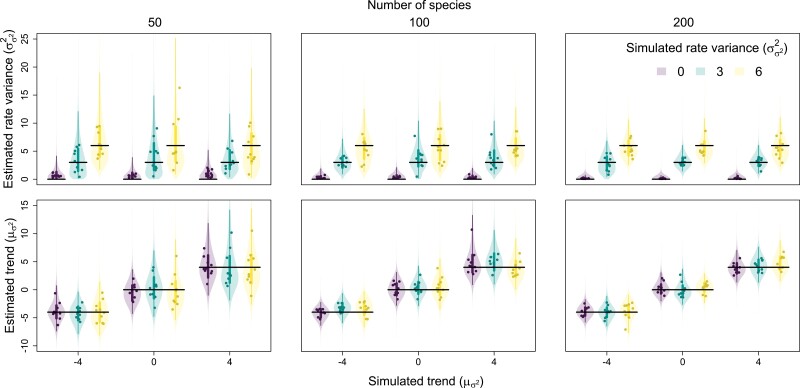
Relationship between simulated and estimated rate variance ($\sigma_{\sigma^{2}}^{2}$) and trend ($\mu_{\sigma^{2}}$) parameters. Each point is the posterior median from a single fit, while the violins are combined posterior distributions from all fits for a given trait evolution scenario. Vertical lines represent the 50% (thicker lines) and 95% equal-tailed intervals (thinner lines) of these combined posteriors, while horizontal lines represent positions of true, simulated values.

Overall, the method exhibited accurate inference and appropriate coverage for all parameters, though posterior breadth was often quite large, especially for trees with 50 tips (Tables 1–3, Fig. 1). Posterior accuracy and precision were highly dependent on trait evolution scenario and tree size. In general, higher values of trends and rate variance were associated with larger posterior MAEs and breadth for their respective parameters, such that increasing trends and high rate variance are estimated with the least accuracy and precision. In some cases, higher trends seemed to increase the MAEs and breadth of rate variance posteriors and vice versa, but this pattern was weak overall. On the other hand, larger tree sizes resulted in smaller posterior MAEs and breadth, such that trees with 200 tips yielded the most accurate, precise posteriors. Coverage for trend and rate variance parameters across all trait evolution scenarios and tree sizes remained consistent at around the theoretical expectation of 95%.

**Table 1:** Median absolute errors of rate variance, trend, and branchwise rate posteriors (i.e., median absolute difference between posterior samples and their true, simulated values, a measure of posterior distribution accuracy), averaged across replicates for each simulated trait evolution scenario and tree size

$\sigma_{\sigma^{2}}^{2}$ =		Rate variance			Trend			Branchwise rates		
		0	3	6	0	3	6	0	3	6
					50 species					
$\mu_{\sigma^{2}}$ =	−4	0.66	1.96	2.55	1.36	1.29	1.83	0.47	0.81	1.00
	0	0.57	2.48	3.69	1.49	2.09	2.45	0.48	0.86	1.06
	4	0.99	1.75	3.00	2.06	2.79	2.91	0.60	0.87	1.01
					100 species					
$\mu_{\sigma^{2}}$ =	−4	0.30	1.01	2.03	0.77	1.08	1.31	0.31	0.73	0.90
	0	0.37	1.62	2.37	1.12	1.20	1.59	0.37	0.76	0.89
	4	0.34	1.56	1.87	1.89	1.63	1.54	0.44	0.83	0.90
					200 species					
$\mu_{\sigma^{2}}$ =	−4	0.13	1.27	1.50	0.77	0.95	1.25	0.24	0.66	0.80
	0	0.11	0.75	1.44	0.92	1.13	0.95	0.23	0.71	0.85
	4	0.18	0.82	1.69	1.00	1.13	1.35	0.27	0.72	0.84

*Note:* ${}^{a}$ $\sigma_{\sigma^{2}}^{2}$ and $\mu_{\sigma^{2}}$ indicate the true, simulated values of rate variance and trend parameters, respectively.

**Table 2:** Breadths of rate variance, trend, and branchwise rate posteriors (i.e., the difference between the 97.5% and 2.5% quantiles of posterior samples, a measure of posterior distribution precision), averaged across replicates for each simulated trait evolution scenario and tree size

		Rate variance			Trend			Branchwise rates		
$\sigma_{\sigma^{2}}^{2}$ =		0	3	6	0	3	6	0	3	6
					50 species					
$\mu_{\sigma^{2}}$ =	−4	3.85	9.07	15.05	5.03	6.08	6.71	2.33	3.17	3.76
	0	3.65	10.07	14.82	5.92	8.26	8.28	2.29	3.41	3.90
	4	4.52	8.66	14.05	10.73	10.75	10.75	3.01	3.49	3.85
					100 species					
$\mu_{\sigma^{2}}$ =	−4	1.56	5.60	8.53	3.27	4.65	4.84	1.66	2.92	3.35
	0	1.91	6.45	9.01	4.31	5.27	6.01	1.87	3.10	3.42
	4	1.69	6.47	8.39	7.61	8.42	7.39	2.06	3.32	3.60
					200 species					
$\mu_{\sigma^{2}}$ =	−4	0.69	4.13	6.43	2.80	3.59	4.01	1.23	2.51	3.06
	0	0.62	4.23	6.21	3.39	3.99	4.06	1.18	2.72	3.23
	4	0.79	3.89	6.14	4.50	5.21	5.65	1.39	2.83	3.22

*Note:* ${}^{a}$ $\sigma_{\sigma^{2}}^{2}$ and $\mu_{\sigma^{2}}$ indicate the true, simulated values of rate variance and trend parameters, respectively.

**Table 3.** Coverage of rate variance, trend, and branchwise rate posteriors (i.e., proportion of times the true, simulated value is greater than the 2.5% posterior distribution quantile and less than the 97.5% quantile) for each simulated trait evolution scenario and tree size

		Rate Variance			Trend			Branchwise Rates		
$\sigma_{\sigma^{2}}^{2}$ =		0	3	6	0	3	6	0	3	6
					50 species					
$\mu_{\sigma^{2}}$ =	−4	—	0.90	1.00	0.80	1.00	1.00	0.98	0.95	0.92
	0	—	0.90	0.90	1.00	0.90	0.80	0.99	0.96	0.92
	4	—	1.00	0.90	1.00	0.90	0.90	0.99	0.96	0.92
					100 species					
$\mu_{\sigma^{2}}$ =	−4	—	1.00	0.90	1.00	1.00	1.00	1.00	0.97	0.92
	0	—	0.80	1.00	1.00	1.00	1.00	0.99	0.96	0.95
	4	—	0.90	1.00	0.90	1.00	1.00	0.97	0.95	0.96
					200 species					
$\mu_{\sigma^{2}}$ =	−4	—	0.90	1.00	1.00	1.00	0.90	1.00	0.94	0.94
	0	—	1.00	1.00	0.90	0.90	1.00	0.99	0.95	0.94
	4	—	1.00	0.90	1.00	1.00	0.90	1.00	0.96	0.95

*Note:* ${}^{a}$ $\sigma_{\sigma^{2}}^{2}$ and $\mu_{\sigma^{2}}$ indicate the true, simulated values of rate variance and trend parameters, respectively.

**Figure 2.**
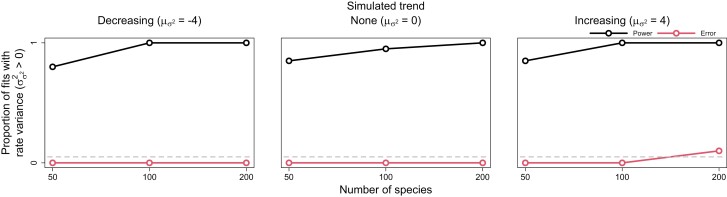
Power and error rates for the rate variance parameter ($\sigma_{\sigma^{2}}^{2}$). Lines depict changes in the proportion of model fits that correctly showed evidence for rate variance significantly greater than 0 (i.e., power, indicated by darker black lines) and incorrectly showed evidence (i.e., error, indicated by lighter red lines) as a function of tree size.

**Figure 3.**
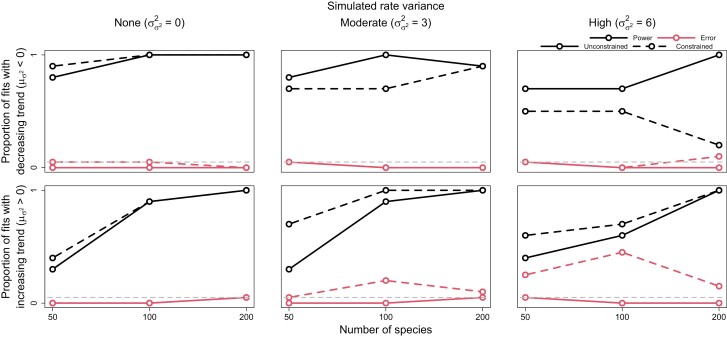
Power and error rates for the trend parameter ($\mu_{\sigma^{2}}$). Lines depict changes in the proportion of model fits that correctly showed evidence for trends significantly less and greater than 0 (i.e., power, indicated by darker black lines) and incorrectly showed evidence (i.e., error, indicated by lighter red lines) as a function of tree size. Results are shown for both models allowed to freely estimate rate variance ($\sigma_{\sigma^{2}}^{2}$) (i.e., unconstrained models, solid lines) and models with rate variance constrained to 0 (i.e., constrained models, dashed lines). The latter models are identical to conventional early/late burst models.

Both the statistical power and error rates of our method were appropriate for detecting trends and significant rate variance. In general, power increased with larger trees, while error rates remained consistent. The ability of SD ratios to identify significant rate variance was particularly impressive, erroneously detecting rate variance only once while exhibiting high power (Fig. 2). Decreasing trends were notably easier to detect than increasing trends, particularly on small trees (Fig. 3). Trend error rates consistently remained below ~5%, and decreasing trends were never mistaken for increasing trends and vice versa. Higher rate variance seemed to only slightly decrease the power to detect trends. Constraining rate variance to 0 resulted in either worse power or higher error rates for detecting trends, depending on whether trends were decreasing or increasing. As rate variance increased, the power of constrained models to detect decreasing trends dramatically diminished. On the other hand, constrained models detected increasing trends with greater power, at the cost of greatly inflated error rates. Overall, estimating rate variance allows for more sensitive detection of declining trait evolution rates while better safeguarding against false detection of increasing rates.

**Figure 4.**
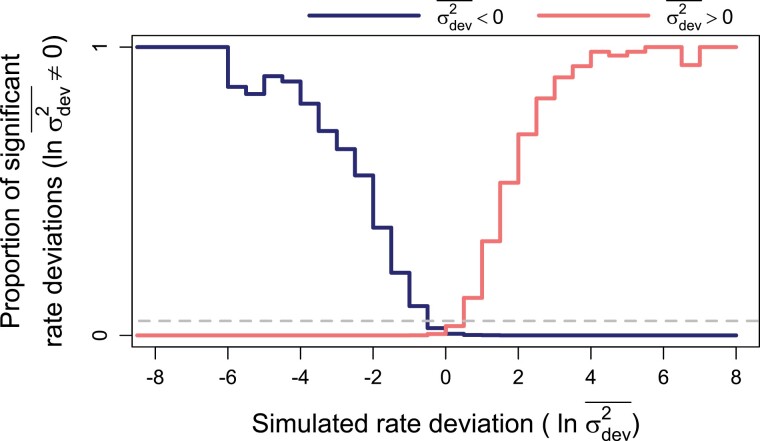
Power and error rates for branchwise rate parameters ($ln\;\bar{\sigma^{2}}$). Lines depict changes in proportions of branchwise rates considered anomalously slow (darker blue line) or fast (lighter red line) as a function of simulated rate deviations ($ln\;\bar{\sigma_{dev}^{2}}$). These results combine all fits to simulated data that detected rate variance ($\sigma_{\sigma^{2}}^{2}$) significantly greater than 0. The proportions are equivalent to power when the detected rate deviation is of the same sign as the true, simulated deviation (left of 0 for anomalously slow rates in darker blue and right for anomalously fast rates in lighter red), and to error rate when the detected and true rate deviations are of opposite signs. Here, significant rate deviations for simulated rate deviations that are exactly 0 are considered errors regardless of sign.

**Figure 5.**
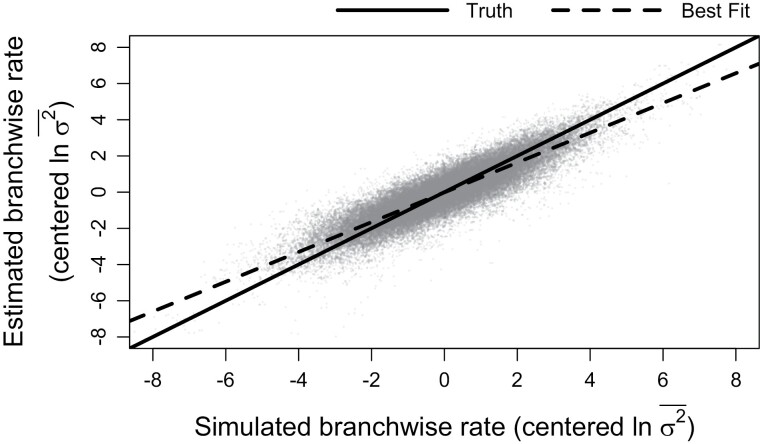
Relationship between simulated and estimated branchwise rate parameters ($ln\;\bar{\sigma^{2}}$). For each simulation and posterior sample, branchwise rates were first centered by subtracting their mean. We estimated centered branchwise rates by taking the median of the centered posterior samples. The solid line represents the position of the true centered branchwise rates, while the shallower, dashed line represents the observed line of best fit for these data.

Branchwise rate estimation also generally displayed appropriate coverage, accuracy, and statistical testing properties (Tables 1–3, Fig. 4). However, branchwise rate estimates were noticeably biased toward their overall mean (i.e., shrinkage). Linear regressions of median branchwise rate estimates on simulated branchwise rates yield an average slope of about 0.8 (Fig. 5). A similar pattern holds for linear regression of branchwise rate deviations (Fig. S1). Branchwise rate posteriors for simulations with no rate variance exhibited especially high accuracy, precision, and coverage (notably above the theoretical expectation of 95%), perhaps due to the increased precision of rate variance posteriors under such trait evolution scenarios. In contrast to other parameters, increasing tree size only slightly decreased posterior MAEs and breadth for branchwise rates. After accounting for variation in simulated branchwise rate deviations, trait evolution scenario and tree size had little effect on statistical power and error rates for detecting anomalous branchwise rates. Averaging across all fits to simulations with significant rate variance detected, error rates for detecting anomalous rates remained negligible, peaking at around 0.5% for branchwise rate deviations of around 0. In fact, this peak only increased to about 5% when we set the significant PP thresholds to 10% and 90% (Fig. S2). The method was somewhat more sensitive to positive than negative deviations, correctly and consistently detecting anomalous rates with deviations more extreme than −4 (1/50th of background rate) or 3 (20 times background rate).

### Empirical Example

**Figure 6.**
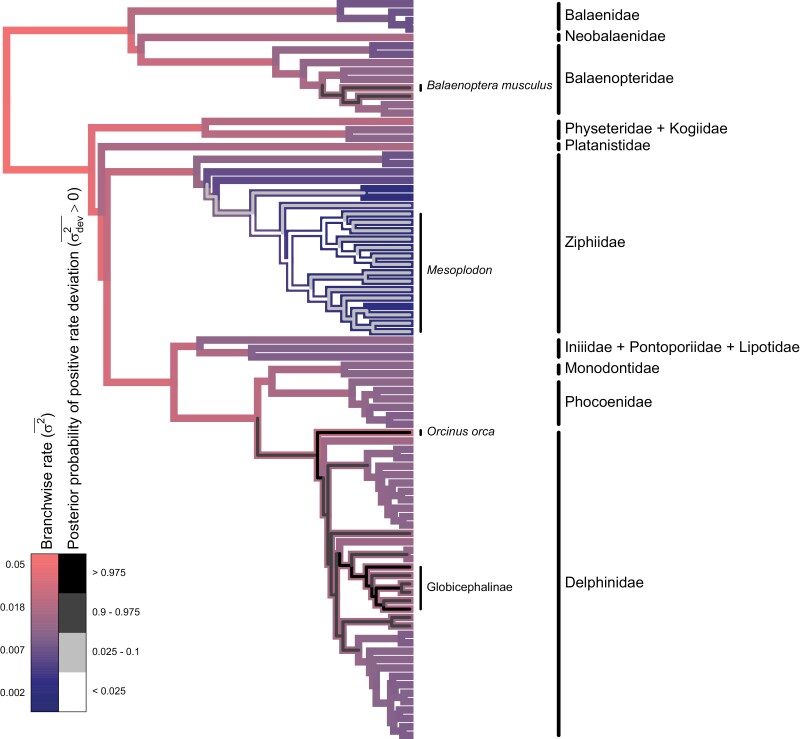
Phylogram of model results for cetacean body size data. Branch colors represent median posterior estimates of branchwise rates ($ln\;\bar{\sigma^{2}}$) of body size evolution, with slower and faster rates in dark blue and light red, respectively. The thinner, inset colors represent the posterior probability that a branchwise rate is anomalously fast according to its rate deviation ($ln\;\bar{\sigma_{dev}^{2}}$), with lower and higher posterior probabilities in light and dark gray, respectively.

**Figure 7.**
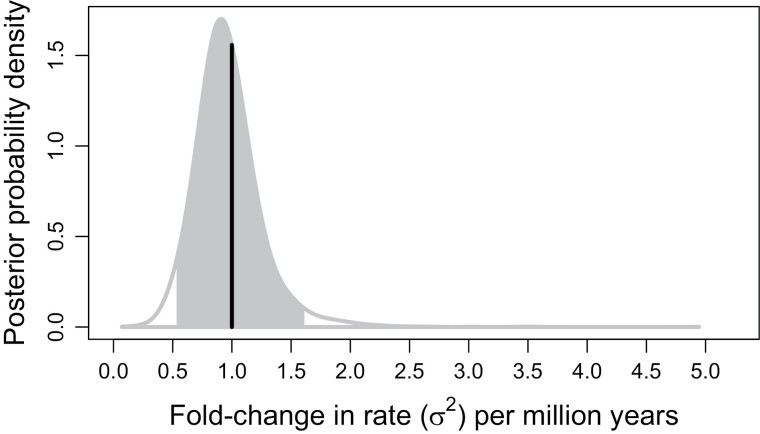
The posterior probability distribution of fold-changes in cetacean body size evolution rates ($\sigma^{2}$) per 1 million years. This distribution is given by $exp\left[\mu_{\sigma^{2}}+\sigma_{\sigma^{2}}X\right]$, where X is a random variable drawn from a standard normal distribution. The gray filled-in portion represents the 95% equal-tailed interval, while the vertical line represents the starting rate of 1.

Overall, our model suggests that rates of body size evolution among extant cetaceans have generally slowed down over time, with considerable divergence in rates of body size evolution among key subclades (Fig. 6). We found marginally significant support for a decreasing trend in rates over time, with rates declining by about 7% every million years (95% CI: 0%–15% decrease, PP of increasing trend: 2.5%). We also infer a moderate rate variance of about 0.06 per million years (CI: 0.01–0.22, SD ratio: 0.14). Combining these two results, changes in body size evolution rates over a million-year time interval are expected to range from a 50% decrease to 60% increase for any particular lineage (Fig. 7).

We also identify a few regions of the cetacean phylogeny where rates of body size evolution seem to be especially low or high. After detrending, rates of body size evolution in the beaked whale genus *Mesoplodon* are about 34% slower than the background rate (CI: 13%–77%, PP of positive rate deviation: <1%). On the other end of the spectrum, some oceanic dolphin lineages exhibit unusually rapid body size evolution rates. In particular, pilot whales and allies (subfamily globicephalinae) and the orca (*Orcinus orca*) lineage exhibit body size evolution rates about 160% (CI: 10%–900%, PP: 99%) and 200% (CI: 20–1,300, PP: 99%) higher than the background rate, respectively. In fact, oceanic dolphins as a whole exhibit a marginally significant increase in body size evolution rates, even after excluding the pilot whale subfamily and orca lineage (CI: 90%–300% background rate, PP: 95%). Similarly, the blue whale (*Balanoptera musculus*) lineage also exhibits a marginally significant increase in body size evolution rate, about 140% (CI: −10% to 1,000%, PP: 95%) higher than the background rate.

Under the model with rate variance constrained to 0, rates of body size evolution decrease by only about 4% every million years (95% CI: −1% to 10% decrease, PP of increasing trend: 7.3%). While only a slight difference, the trend parameter estimated under the full model yields a marginally significant, two-tailed “*p*-value” of ~5%, while the constrained model yields a decidedly insignificant “*p*-value” of ~15%. This is reflected in a conventional sample-size corrected Akaike Information Criterion (AICc) comparison between simple BM and EB models of trait evolution fitted via maximum likelihood (ML) using the R package *geiger* (version 2.0.7; Pennell et al., 2014). In this case, a simple BM model receives nearly twice the AICc weight of an EB model (65% vs. 35%).

## Discussion

Here we implemented a novel data-driven method, *evorates*, for modeling stochastic, incremental variation in trait evolution rates. Part of the power of *evorates* is its ability to infer trait evolution rate variation independent of an *a priori* hypothesis on what factors influence rates. This allows for detailed, hypothesis-free exploration of trait evolution rate variation across time and taxa. Researchers may use such results to generate and refine hypotheses regarding what factors have influenced trait evolution rates across the tree of life (e.g., Uyeda et al., 2018). Overall, *evorates* performs well on simulated data, recovering accurate parameter estimates and exhibiting appropriate statistical power and error rates for hypothesis testing. Further, the method shows great promise for empirical macroevolutionary research, offering novel insights into the dynamics of cetacean body size evolution—a notably well-studied system (e.g., Slater et al., 2010, Pyenson and Sponberg, 2011, Montgomery et al., 2013, Slater and Pennell, 2014; Slater et al., 2017; Sander et al., 2021). The results of our study also build on previous work in demonstrating that estimating time-independent rate heterogeneity is critical for accurately quantifying temporal dynamics in trait evolution rates (Slater and Pennell, 2014). This finding has consequences for how EBs/LBs of trait evolution are practically identified and conceptually defined.

The simulation study results showcase *evorate’s* ability to recover accurate parameter estimates across a range of tree sizes. Despite the high uncertainty of rate variance estimates under some trait evolution scenarios, rate heterogeneity could still be correctly detected about 90% of the time with an error rate substantially lower than 5%. Indeed, our hypothesis testing procedures seem conservative in general, exhibiting relatively low error rates. While it could be beneficial to relax significance thresholds for SD ratios and/or posterior probabilities for increased statistical power, our hypothesis testing procedures seem sufficiently powered and we thus do not explore alternative thresholds in great detail here (but see Fig. S2). In any case, compared to conventional EB/LB models, *evorates* can detect decreasing trends in trait evolution rates with greater sensitivity and detect increasing trends with greater robustness. Notably, traits evolving with exponentially increasing rates on an ultrametric phylogeny (i.e., an LB model) exhibit the same probability distribution expected under a single-peak Ornstein-Uhlenbeck (OU) model, where traits evolve toward some optimum at a constant rate (Blomberg et al., 2003). Therefore, the frequently observed support for single-peak OU models from ultrametric comparative data (e.g., Harmon et al., 2010; see also Cooper et al., 2016; Landis and Schraiber, 2017) may partially result from autocorrelated rate heterogeneity, which inflates support for LB/OU models based on our simulation study. Despite their mathematical similarities, LB, OU, and our new models have distinct biological interpretations regarding the importance of rate heterogeneity and selective forces in shaping the patterns of trait diversity within clades.

Interestingly, closer inspection of our simulation study results suggests that, in the presence of rate heterogeneity, models with rate variance constrained to 0 (i.e., conventional EB/LB models) estimate trend parameters corresponding to changes in average trait evolution rates over time. On the other hand, unconstrained *evorates* models estimate trend parameters corresponding to changes in median trait evolution rates over time, essentially determining whether most lineages in a clade exhibit rate decreases or increases (Figs. S19–S21; Tables S5–S7). Counterintuitively, when the trend parameter is only weakly negative relative to rate variance ($-\sigma_{\sigma^{2}}^{2}/2<\mu_{\sigma^{2}}<0$), it is possible for a majority of lineages within a clade to exhibit declining trait evolution rates (i.e., an EB according to *evorates*) while rates averaged across the entire clade increase over time (i.e., an LB according to conventional methods). This occurs because rates evolve in a right-skewed manner under our model—in other words, a few anomalous lineages/subclades tend to evolve extremely high-trait evolution rates in spite of declining rates among most other lineages, driving up a clade’s overall average rate (Figs. S17–S18). We note that *evorates* still returns estimates of average changes in trait evolution rates per unit time via a simple parameter transformation ($\mu_{\sigma^{2}}+\sigma_{\sigma^{2}}^{2}/2$). We choose to focus on the majority-based definition of EBs/LBs since, by accounting for anomalous lineages/subclades exhibiting unusual rates, this definition better matches many macroevolutionary biologists’ intuitive definition of EBs (Lloyd et al., 2012; Slater and Pennell, 2014; Benson et al., 2014; Hopkins and Smith, 2015; Wright, 2017; Puttick, 2018).

Our empirical example with cetacean body size directly demonstrates the practical importance of these nuances in defining EB/LB dynamics. We find substantial evidence that body size evolution has slowed down in most cetacean lineages, despite the presence of “outlier” lineages exhibiting relatively rapid rates. Indeed, we find little evidence for a decline in body size evolution rates averaged across the clade (95% CI: 12% decrease − 5% increase in average rate per million years, PP of increasing average rate: 16%). This broadly agrees with previous research, but *evorates* is able to offer novel insights and contextualize prior results by explicitly estimating branchwise rates in addition to overall trends (Slater and Pennell, 2014; Sander et al., 2021). For example, Slater and Pennell (2014) identified the orca and pilot whale lineages as outlier lineages exhibiting especially rapid rates of body size evolution. Our method recapitulates these findings while suggesting oceanic dolphins as a whole represent a relatively recent burst of body size evolution that has largely masked signals of an earlier burst toward the base of the clade. Such findings more generally agree with recent suggestions that bursts of trait evolution may be common but not limited to the base of “major” clades. This is likely due, in part, to major clades being arbitrarily designated based on taxonomic rank (Puttick, 2018). Alternatively, some propose that EBs may be hierarchical, with major clades exhibiting repeated bouts of rapid trait diversification as competing, closely related lineages partition niche space more finely over time (Slater and Friscia, 2019). Ultimately, we are optimistic that *evorates* may be better able to resolve how frequently bursts of trait evolution—early or not—occur across the tree of life compared to more conventional methods.

The shrinkage of branchwise rates, whereby rate estimates are biased toward their overall mean, is presumably due to the assumption that rates are autocorrelated under our model. Because of this, rate estimates are partially informed by the rates in closely related lineages, particularly when closely related lineages are better sampled (i.e., more related to taxa with sampled trait values and/or consisting of many short branch lengths). This “diffusion” of rates across the phylogeny appears to cause under- and overestimation of unusually high and low rates, respectively. Fortunately, this renders *evorates* conservative in terms of identifying anomalous trait evolution rates, safeguarding against erroneous conclusions. In general, we view this behavior as a good compromise between model flexibility and robustness, allowing *evorates* to infer rate variation while avoiding ascribing significance to noise in data. We note that rate variance estimates under our model are largely unbiased, such that branchwise rates in a typical posterior sample should be as variable as the true rates. Thus, taking the joint distribution of branchwise rates into account by analyzing distributions of *differences* between rates, rather than just assessing marginal distributions of rates, appears important in accurately interpreting results under our model. In any case, despite this shrinkage phenomenon, the statistical power to identify overall rate heterogeneity and anomalous rates with *evorates* appears comparable to that of previous data-driven methods (Eastman et al., 2011).

*Evorates* is one of several recently developed methods that also estimate unique trait evolution rates for each branch in a phylogeny but assume an alternative mode of rate change (May and Moore, 2020; Fisher et al., 2021). These other methods assume that branchwise rates are independently distributed according to a log-normal distribution. The method we develop here differs from these “independent rate” (IR) models in assuming that rates evolve gradually and are thus phylogenetically autocorrelated (see also Revell, 2021). Theoretically, trait evolution rates should exhibit some degree of phylogenetic autocorrelation given that many factors hypothesized to affect trait evolution rates themselves exhibit phylogenetic autocorrelation. Indeed, a recent study found evidence for autocorrelation of trait evolution rates in a few vertebrate clades (Sakamoto and Venditti, 2018), and autocorrelation has also been found in lineage diversification (Savolaine et al., 2002; Caron and Pie, 2020) and molecular substitution rates (Lepage et al., 2006; Tao et al., 2019). Notably, there is also no known rate evolution process that would produce independent, log-normally distributed branchwise rates (Lepage et al., 2006, 2007). However, IR models could outperform “autocorrelated rate” (AR) models in some instances due to their tremendous flexibility in modeling how rates vary over time and phylogenies. In general, we expect that IR models will perform best in cases with many traits and/or non-ultrametric trees, where the flexibility of the model can be tempered by rich information content in the data. More work testing for rate autocorrelation or lack thereof in continuous trait data is needed as methods for inferring trait evolution rate variation become more complex.

Revell (2021) independently developed a method, *multirateBM*, based on a model similar to the one we introduce here, though *evorates* offers several key advantages. In particular, the ML implementation of *multirateBM* renders it impossible to estimate rate variance. To do so, one would need to analytically marginalize over uncertainty in branchwise rates. Here, we circumvent this issue by using Bayesian inference to numerically integrate over uncertainty in branchwise rates. This is analogous to how ML implementations of mixed effect models analytically marginalize over uncertainty in random effects, while Bayesian implementations of the same models sample random effects (Browne and Draper, 2006). Indeed, ML implementations of mixed effect models that treat random effects as parameters would be unable to estimate random effect variances due to the very same reasons *multirateBM* cannot estimate rate variance. Additionally, our model has the added advantage of accommodating both trends in rates and uncertainty in tip trait values. Lastly, we implement procedures to test the significance of rate heterogeneity, trends, and anomalous trait evolution rates. While *multirateBM* offers a quick and convenient means for comparative data exploration, our new method allows for more rigorous quantification and analysis of rate evolutionary processes and patterns from comparative data.

There are a number of ways the *evorates* might be improved or expanded. Assuming that trait evolution rates for different traits are correlated with one another, using data on multiple traits could improve inference of both the rate evolution process and branchwise rate parameters (May and Moore, 2020). Another promising future direction is integration of *evorates* with hypothesis-driven methods. This could be done *post hoc* by applying phylogenetic linear regression to “tip rates” estimated under the model (e.g., Rabosky and Huang, 2016) or analyzing distributions of branchwise rates associated with ancestral states estimated via stochastic character maps (Revell, 2013; but see May and Moore, 2020). Alternatively, one could explicitly model rates as the product of both a stochastic rate evolution process and a deterministic function of some factor of interest. We have already taken steps toward this model extension in our current implementation by allowing rates to change as a deterministic function of time. Lastly, despite our focus on gradually changing rates, trait evolution rates might also exhibit sudden shifts of large magnitude (“jumps”) or short-lived fluctuations (“pulses”) in response to factors with a particularly strong influence on rates. It would be ideal—but difficult—to model rates as evolving gradually, while potentially undergoing sudden jumps or pulses (e.g., Lartillot et al., 2016). An alternative strategy is developing methods to compare the fit of a model like ours against more conventional data-driven models whereby rates jump or even Lévy models whereby rates pulse (Landis et al., 2013). Assessing when and whether comparative data can distinguish between different modes of rate change will be important for future research on the dynamics of trait evolution.

## Conclusion

Here, we introduced *evorates*, a method that models gradual change, rather than abrupt shifts, in continuous trait evolution rates from comparative data. Unlike nearly all other comparative methods for inferring rate variation, *evorates* goes beyond identifying lineages exhibiting anomalous rates by also estimating the process by which rates themselves evolve. Although there are many potential modes of rate variation over time and phylogenies, our model estimates rate evolution processes as the product of two parameters: one controlling how quickly rates accumulate random variation, and another determining whether rates tend to decrease or increase over time. The resulting method returns accurate estimates of evolutionary processes and provides a flexible and intuitive means of detecting and analyzing trait evolution rate variation. Looking forward, *evorates* has tremendous potential for improvement and elaboration, and we are optimistic that the future of macroevolutionary biology will benefit from increased focus not only on how traits evolve, but how the rates of trait evolution themselves evolve over time and taxa.

## SUPPLEMENTARY MATERIAL

Data available from the Dryad Digital Repository: http://dx.doi.org/10.5061/dryad.9ghx3ffkb. The current version of the *evorates* R package is available at the GitHub repository: https://github.com/bstaggmartin/evorates.
